# Mesenchymal Stromal Cells Promote Retinal Vascular Repair by Modulating Sema3E and IL-17A in a Model of Ischemic Retinopathy

**DOI:** 10.3389/fcell.2021.630645

**Published:** 2021-01-21

**Authors:** Baraa Noueihed, José Carlos Rivera, Rabah Dabouz, Pénélope Abram, Samy Omri, Isabelle Lahaie, Sylvain Chemtob

**Affiliations:** ^1^Department of Ophthalmology, Maisonneuve-Rosemont Hospital Research Center, University of Montréal, Montréal, QC, Canada; ^2^Department of Pharmacology and Therapeutics, McGill University, Montréal, QC, Canada; ^3^Departments of Pediatrics, Ophthalmology and Pharmacology, Centre Hospitalier Universitaire Sainte-Justine Research Center, Montréal, QC, Canada

**Keywords:** interleukin-17A, semaphorin 3E, ischemic retinopathies, vascular regeneration, mesenchymal stem cells

## Abstract

Ischemic retinopathies (IRs), such as retinopathy of prematurity and diabetic retinopathy, are characterized by an initial phase of microvascular degeneration that results in retinal ischemia, followed by exaggerated pathologic neovascularization (NV). Mesenchymal stromal cells (MSCs) have potent pro-angiogenic and anti-inflammatory properties associated with tissue repair and regeneration, and in this regard exert protection to neurons in ischemic and degenerative conditions; however, the exact mechanisms underlying these functions remain largely unknown. Class III Semaphorins (A–G) are particularly implicated in regulating neural blood supply (as well as neurogenesis) by suppressing angiogenesis and affecting myeloid cell function; this is the case for distinct neuropillin-activating Sema3A as well as PlexinD1-activating Sema3E; but during IR the former Sema3A increases while Sema3E decreases. We investigated whether retinal vascular repair actions of MSCs are exerted by normalizing Semaphorin and downstream cytokines in IR. Intravitreal administration of MSCs or their secretome (MSCs-conditioned media [MSCs-CM]) significantly curtailed vasoobliteration as well as aberrant preretinal NV in a model of oxygen-induced retinopathy (OIR). The vascular repair effects of MSCs-CM in the ischemic retina were associated with restored levels of Sema3E. Vascular benefits of MSCs-CM were reversed by anti-Sema3E; while intravitreal injection of anti-angiogenic recombinant Sema3E (rSema3E) in OIR-subjected mice reproduced effects of MSCs-CM by inhibiting as expected preretinal NV but also by decreasing vasoobliteration. To explain these opposing vascular effects of Sema3E we found in OIR high retinal levels, respectively, of the pro- and anti-angiogenic IL-17A and Sema3A-regulating IL-1β; IL-17A positively affected expression of IL-1β. rSema3E decreased concentrations of these myeloid cell-derived pro-inflammatory cytokines *in vitro* and *in vivo*. Importantly, IL-17A suppression by MSCs-CM was abrogated by anti-Sema3E neutralizing antibody. Collectively, our findings provide novel evidence by which MSCs inhibit aberrant NV and diminish vasoobliteration (promoting revascularization) in retinopathy by restoring (at least in part) neuronal Sema3E levels that reduce pathological levels of IL-17A (and in turn other proinflammatory factors) in myeloid cells. The ability of MSCs to generate a microenvironment permissive for vascular regeneration by controlling the production of neuronal factors involved in immunomodulatory activities is a promising opportunity for stem cell therapy in ocular degenerative diseases.

## Introduction

Ischemic retinopathies (IRs), such as retinopathy of prematurity (ROP) and diabetic retinopathy (DR), are leading causes of severe visual impairment and blindness in children and the working population, respectively, (Mora et al., 2018; Sabanayagam et al., 2019). These ocular diseases are characterized by retinal vasculature impairment that originates from local hypoxia, which triggers an exaggerated and uncontrollable pathological neovascularization (NV). Several molecules have been described to play an important role in pathological ocular NV, of which vascular endothelial growth factor (VEGF) is most reported. Anti-VEGF therapy has proven effective in reducing NV areas and improving the vision of some patients with retinopathy. However, anti-VEGF agents do not facilitate revascularization of the retina, resulting in undesired outcome (Ghasemi Falavarjani and Nguyen, 2013; Bracha et al., 2018; Low et al., 2019). Lately, adult stem-cell based therapies have emerged as alternative therapeutic avenues to treat IRs (Dorrell et al., 2004; Sivan et al., 2016; Bertelli et al., 2020). In particular, Mesenchymal Stem/Stromal Cells (MSCs) are a most promising modality for the treatment of ocular diseases (Joe and Gregory-Evans, 2010; Ding et al., 2017) because of their remarkable immunomodulatory and angiogenic capacities (Singer and Caplan, 2011; Nassiri and Rahbarghazi, 2013; Tao et al., 2016; Weiss and Dahlke, 2019); correspondingly, MSCs exert protective functions on brain and retinal neurons (Harrell et al., 2019; Zhang et al., 2020). These adult multipotent stromal cells exert their reparative effects either by cell differentiation to replace damaged cells or through paracrine fashion via their secretome (Konala et al., 2016; Jaimes et al., 2017; Mead and Tomarev, 2017; Harrell et al., 2018); however, since MSCs have low engraftment efficiency and poor differentiation at the site of injury, an action involving secretion of bioactive molecules seems to be favored in their modulation of tissue microenvironment (Tran and Damaser, 2015; Harrell et al., 2019). But, the exact mechanism of MSC functions on vascular repair is likely diverse and remains largely unclear.

We previously highlighted the importance of neuron-derived signaling molecules on retinal endothelial cell function (Joyal et al., 2011; Rivera et al., 2013), particularly as it applies to guidance molecules of the class III semaphorins family (Sema3A-Sema3G), which are derived from retinal ganglion cells (RGCs), and are key regulators of developmental and pathological angiogenesis in the retina (Fukushima et al., 2011; Joyal et al., 2011; Kim et al., 2011; Buehler et al., 2013; Yang et al., 2015; Ochsenbein et al., 2016). Semaphorins can in turn affect the activity of myeloid cells (Ito et al., 2012; Neufeld et al., 2016). Most Sema3s (as clearly described for Sema3A-E) exert anti-angiogenic functions (Toledano et al., 2019). Interestingly, Sema3E (a key axon repulsive protein) is implicated in immune cell regulation and vascular growth and remodeling (Oh and Gu, 2013; Shimizu et al., 2013; Wanschel et al., 2013; Movassagh et al., 2017b; Alamri et al., 2018; Kermarrec et al., 2019), as it selectively inhibits disoriented outgrowth of extraretinal vessels and restores the normal vasculature in ischemic retina (Fukushima et al., 2011). Sema3E exerts its actions on endothelial cells via a single-pass transmembrane receptor, PlexinD1 (Kruger et al., 2005). During retinal development as well as in ischemic retinopathy, PlexinD1 is located on endothelial cells exclusively at the front of actively sprouting blood vessels, while its ligand Sema3E is generated by RGCs (Kim et al., 2011; Sun et al., 2017). Sema3E-PlexinD1 complex trigger an antiangiogenic signaling pathway that suppresses endothelial cell motility by inducing cytoskeletal rearrangements that cause filopodial retraction (Sakurai et al., 2010; Fukushima et al., 2011), and inhibits endothelial cell growth and tube formation (Kim et al., 2011). Semaphorins can also modulate angiogenesis (and tumor progression) by regulating immune cell function, as documented for Sema3A and Sema3E (Sierra et al., 2008; Movassagh et al., 2019). Conversely, decreased expression of Sema3E aggravates inflammation and exacerbates disease severity by upregulating the release of pro-inflammatory cytokines, notably of interleukin-17A (IL-17A) (Movassagh et al., 2017b; Kermarrec et al., 2019), which in turn can affect NV (Talia et al., 2016; Li and Zhou, 2019); concordantly, IL-17A plasma levels are high in DR and ROP (Sood et al., 2010; Semeran et al., 2013). Hence, Semaphorins seem to represent an important class of factors that exert important vascular, immuno-modulatory and neuronal functions. However, the role of Sema3E and its interaction with IL-17A as it applies to MSC actions in IRs, is not known.

Using an OIR mouse model, we showed that MSCs modulate the microenvironment of ischemic retinas enabling vascular regeneration and thus diminishing vasoobliteration and inhibiting aberrant pre-retinal NV. MSCs secretome suppresses the expression of Sema3A and intriguingly markedly stimulates that of Sema3E, both of which arise from the ganglion cells in retina of animals subjected to OIR. Upon exploring the role of Sema3E it has been found that this Semaphorin not only inhibits NV by acting directly on vascular endothelium (Toledano et al., 2019) but also acts on myeloid cells by repressing pro-angiogenic IL-17A expression (Li and Zhou, 2019), that negatively impacts production of primary pro-inflammatory cytokines IL-1β, IL-6, and TNF-α in these cells; these cytokines are known to upregulate expression of the anti-angiogenic Sema3A and conversely its suppression enhances retinal revascularization of vasoobliterated areas (Joyal et al., 2011; Rivera et al., 2013). Thus, in presence of MSCs, we hereby find that Sema3E mediates to a significant extent the vascular benefits incurred by MSCs secretome, and assists Sema3A to performing opposing functions, specifically accelerating revascularization and diminishing aberrant NV, by controlling inflammation. Collectively, we highlight some evidence by which MSCs secretome regulates the interplay between neurons and immune cells to facilitate healthy revascularization and diminish abnormal NV in ischemic retina.

## Materials and Methods

### Animals

Adult C57BL6/J mice were purchased from Jackson Laboratories. Adoptive lactating CD-1 females were purchased from Charles River to tend to C57BL6/J pups. All experiments adhered to the Association for Research in Vision and Ophthalmology (ARVO) statement regarding use of animals in ophthalmic and vision research and were approved by the Animal Care Committee of Maisonneuve-Rosemont Hospital in accordance with guidelines established by the Canadian Council on Animal Care.

### Isolation and Characterization of Mesenchymal Stem/Stromal Cells From Compact Bone

MSCs characterized by their potential therapeutic properties, easy isolation, wide expansion *in vitro*, and their abundance in compact bone of many species (Adhikari et al., 2018; Yusop et al., 2018) were isolated from the compact bone of mice as previously outlined (Zhu et al., 2010). Long bones of adult C57BL6/J mice (6–8 weeks) were removed and thoroughly cleaned from any connective tissue. Using a 23G needle, the bone marrow was flushed out with PBS and saved for subsequent isolation of bone-marrow-derived macrophages (BMDM). Bones were gently crushed and digested with (2.5 mg/ml) collagenase (Sigma) and 5% TrypLE Express (Gibco) for 1 h at 37°C. Cell suspension was discarded, and bone fragments were cultivated in αMEM (Gibco) supplemented with 20% Mesenchymal-tested FBS (Wisent) and 1% penicillin/streptomycin (Corning) at hypoxia (5%O_2_) allowing the migration of MSCs out of the compact bone. MSCs were enriched at P2 by negative selection using EasySep Mouse Mesenchymal Progenitor Enrichment Kit (Stem Cell Technologies) to deplete non-mesenchymal lineages. At 80% confluency, the media was changed to basal αMEM. The supernatant, referred herein as conditioned media (MSCs-CM), was collected 24 h later, centrifuged, filtered through 0.22 μm filter (Millipore), and concentrated 10 times using a 10 K molecular weight cut off centrifugal filter (Millipore). MSCs-CM was collected between passages 3 and 5. MSCs were characterized based on the minimal criteria put forth by the International Society of Cellular Therapy (Dominici et al., 2006). Immunophenotyping of MSCs was performed by flow cytometry analysis using monoclonal conjugated antibodies against putative MSC surface markers FITC anti-mouse CD90.2 (eBioscience), PE/Cy7 anti-mouse CD105 (BioLegend), PE anti-mouse CD73 (BioLegend), and against cell lineage markers FITC anti-mouse CD11b (eBioscience) and PE anti-mouse CD34 (BioLegend). FACS was performed on BD LSRFortessa X-20 and data was analyzed using FlowJo software. Compact bone derived-MSCs highly expressed the classical stem cells surface makers CD90.2 (≥97%), CD73 (≥94%), and CD105 (≥95%) previously characterized in C57BL6/J mice (Caroti et al., 2017) and were negative for hematopoietic and myeloid markers including CD11b (≤1%) and CD34 (≤0.3%) (Supplementary Figures 1A,B). MSCs were also differentiated in adipocytes or osteocytes following the instructions described in the mouse Mesenchymal Stem Cell Functional Identification Kit (RnD systems). MSCs’ adipogenic and osteogenic potential was confirmed by immunostaining against fatty acid binding protein 4 (FABP4) and osteopontin, respectively, (Supplementary Figure 1C).

### Isolation and Stimulation of Bone-Marrow Derived Macrophages

Bone marrow from tibia and femur of adult mice were flushed with PBS and gently dissociated with 23G needle. The cell suspension was filtered through 70 μm strainer and centrifuge at 4°C, 500 × *g* for 10 min. Cell pellet was resuspended in DMEM containing 10% FBS and 20 ng/ml monocyte colony stimulating factor (M-CSF; PeproTech) to differentiate monocytes into macrophages and seeded in six-well plates. On days 3 and 5, half of the differentiation media was changed. By day 7, mature macrophages have formed and attached to the plate. Bone-marrow derived macrophages (BMDM) then were pre-exposed to hypoxia for 24 h to assess IL-17A expression levels. Subsequently, BMDM were treated with vehicle, MSCs-CM, HypRGC-CM, HypRGC-MSCs-CM, and HypRGC-MSCs in presence of a monoclonal Sema3E antibody (15 μg/ml; R&D Systems), recombinant mouse Sema3E (5 ng/ml), or recombinant mouse IL-17A (100 ng/ml) proteins for another 24 h. RNA was isolated using RNA isolation kit (Qiagen), whereas protein was collected in commercial RIPA buffer (Cell Signaling Technology).

### Oxygen-Induced Retinopathy Model

The OIR model has been well established and standardized to mimic the vascular dysfunction observed in ROP (Smith et al., 1994; Stahl et al., 2010). Postnatal day 7 (P7) C57BL6/J pups were placed with an adoptive lactating CD-1 female in a hyperoxic environment set to 75% O_2_ (OxyCycler A820CV; BioSpherix, Ltd., Redfield, NY, United States) until P12 to trigger vaso-obliteration (VO). Then, the animals were returned to room air where by hypoxia-driven NV develops at P14 and reaches its peak at P17. Pups at P17 were anesthetized in 3% isoflurane with oxygen and sacrificed by decapitation. Eyes were enucleated and fixed in 4% paraformaldehyde for 1 h at room temperature. Retinas were dissected and stained overnight at 4°C with fluorescein labeled GSL I, Isolectin B4 (Vector Labs, 1:100) in PBS containing 1 mM CaCl_2_. Lectin-stained retinas were whole-mounted onto Superfrost/Plus microscope slides (Fisher Scientific) with the photoreceptor side down and embedded in Fluoro-gel (Electron Microscopy Sciences). Multiple 10× images of each whole-mounted retina were taken using the MosiaX option built in Zeiss AxioObserver.Z1 and then merged into a single file using the in the AxioVision 4.6.5 software (Zeiss). Quantification of VO and NV was determined at P17 using SWIFT_NV as described previously (Stahl et al., 2009).

### Stimulation of RGC-5

RGC-5 cells were cultured in DMEM (Invitrogen) supplemented with 10% FBS (Cell Applications) and 1% penicillin/streptomycin (Cell Applications) either in normoxia or hypoxia (5% O_2_) and treated with MSCs-CM for 48 h to determine Sema3E mRNA levels. Normoxic and hypoxic RGC-5 cells were stimulated with recombinant IL-17A in a dose-dependent manner for 24 h. RNA was collected in Ribozol (Amersco), whereas protein was collected in commercial RIPA buffer (Cell Signaling Technology).

### Immunohistochemistry

Eyes of P17 C57BL6/J pups exposed to normoxia or OIR were enucleated, fixed in 4% paraformaldehyde for 1 h at room temperature, and saturated overnight at 4°C in a 30% sucrose solution prior to embedding in OCT compound (TissueTek^®^). Sagittal cross-sections of 10 μm was sectioned using a Cryostat (Leica) and permeabilized for 1 h at room temperature. Immunostaining against Sema3E (R&D Systems; 1:100), NeuN (EMD Millipore; 1:100), F4/80 (Abcam; 1:100), or IL-17A (Abcam; 1:200) overnight at 4°C, followed by fluorochrome-conjugated secondary antibody (goat anti-mouse IgG Alexa Fluor 488 and goat anti-rabbit IgG Alexa Fluor 594; Invitrogen) for localization studies according to manufacturers’ recommendations. Nuclei were stained with DAPI (Invitrogen; 1:5000). Cross-sections were visualized using 30× objectives with an IX81 confocal microscope (Olympus), and images were obtained with Fluoview 3.1 software (Olympus).

### Retinal Whole-Mounts

Mice eyes were collected and processed for retinal flat-mounts as previous published studies (Sitaras et al., 2015; Rivera et al., 2017b). Briefly, eyes were fixed in 4% paraformaldehyde for 1 h and then, retinas were isolated and incubated overnight at 4°C in a solution containing 1% Triton X-100-1 mM CaCl_2_/phosphate-buffered saline (PBS) and TRITC-conjugated lectin endothelial cell marker Bandeiraea simplicifolia (1:100; Sigma-Aldrich, St. Louis, MO, United States). Retinas were mounted and photographed under a Zeiss AxiObserver Z1 motorized inverted microscope (Zeiss, Canada) at 10×. Vaso-obliterated areas were assessed as the retinal area devoid of vasculature over the total retinal area by using Adode Photoshop CS5 (Connor et al., 2009). NV was analyzed using the SWIFT-NV method (Stahl et al., 2009) which was developed to quantify all the pixels represented by neovascular tufts and clusters, but not normal vessels in lectin-stained retinal whole mounts.

### Intravitreal Injections

P12 mice pups were anesthetized in 3% isoflurane with oxygen and injected intravitreally with 2 μl of CM, whereas basal αMEM (vehicle) was injected in the contralateral eye as control using a Hamilton syringe equipped with 50-gage glass capillary. 2 μl of mouse recombinant Sema3E Fc Chimera (20 ng/μl; R&D systems) or PBS were administered into vitreous cavity of P12 OIR pups. To assess the effect of blocking IL-17A on the retinal vascularization, 2 μl of 5 μg/μl neutralizing monoclonal IL-17A antibody (Clone 17F3, BioXCell) or of 5 μg/μl mouse IgG1 isotype control monoclonal antibody (Clone MOPC-21, BioXCell) was intravitreally injected. To reverse the benefits of Sema3E induced by MSCs-CM administration during OIR, the eyes were injected at P12 and P14 with 2 μl of blocking Sema3E (5 μg/μl) antibody (AF3239, R&D Systems). To assess the dose-response of MSCs on vasoobliteration, 50,000, 100,000, and 200, 000 cells from passages 3–5 were intravitreally injected in a volume of 2 μl. Retinal vasculature was analyzed in whole-mounts at P17.

### Reverse-Transcription PCR and Quantitative Real-Time PCR

Freshly dissected retinas (2 retinas pooled per n) were placed immediately in RiboZol (Amresco) and homogenized using Precellys 24 homogenizer. Cultured cells were scrapped using RiboZol (Amresco). RNA was extracted following manufacturer’s instructions and then treated with DNase I (Sigma) to remove genomic DNA. 1 μg of RNA was reverse transcribed into cDNA using iScript RT Supermix (Biorad) as described by manufacturer’s instructions. cDNA was analyzed by Quantitative real-time PCR using iTaq^TM^ Universal SYBR^®^ Green Supermix (BioRad) with primers targeting mouse Sema3E (Fwd 5′-TCTGCAACCCATCCATTCTGAG-3′ and Rev 5′-ACCACAAG AGGGAAGCACAGAC-3′), mouse IL-1β (Fwd 5′-CTGGTAC ATCAGCACCTCACA-3′ and Rev 5′-GAGCTCCTTAAC ATGCCCTG-3′), mouse IL6 (Fwd 5′-ACAGAAGGAGTGGCTA AGGA-3′ and Rev 5′-AGGCATAACGCACTAGGTTT-3′), mouse TNF-α (Fwd 5′-GCCTCTTCTCATTCCTGCTTG-3′ and Rev 5′-CTGATGAGAGGGAGGCCATT-3′), mouse IL-17A (Fwd 5′-CACCGCAATGAAGACCCTGA-3′ and Rev 5′-TTCCC TCCGCATTGACACAG-3′), mouse Rorγ (Fwd 5′-CCACT ACGGGGTTATCACCTG-3′ and Rev 5′-TGCAGGAGTAG GCCACATTAC-3′), mouse PlexinD1 (Fwd 5′-TTCCATT TGGTGCTACCTACG-3′ and Rev 5′-CAATACTTTCTTGC GGTGGC-3′), mouse Sema3A (Fwd 5′-GGGGAACCAGATGA CAGAGA-3′ and Rev 5′-GCTCCTGCTCCGTAGCCTGC-3′), mouse VEGF (Fwd 5′-TCGGCGTTGCTTTCGGTCCC-3′ and Rev 5′-GCCCTGAGTCAAGAGGACAG-3′), mouse IL-4 (Fwd 5′-CCATATCCACGGATGCGACA-3′ and Rev 5′-CG TTGCTGTGAGGACGTTTG-3′), mouse IL-10 (Fwd 5′-GC TCTTACTGACTGGCATGA-3′ and Rev 5′-AGAAAGTCTTC ACCTGGCTG-3′). Primers were designed by NCBI Primer-BLAST software and synthesized by Alpha DNA (Montreal). Quantitative gene expression analysis was evaluated using ABI 7,500 Real-Time PCR system (Applied Biosystems) and normalized to QuantumRNA^TM^ 18S universal primer (Invitrogen) using ΔΔCT method.

### Western Blot

Cultured cells were washed with ice-cold PBS, collected in RIPA lysis buffer (Cell Signaling Technology) and centrifuged to eliminate cell debris. 50 μg of cell lysate was loaded on SDS-PAGE gel. After blocking, membranes were blotted against mouse IL-17A (Abcam), IL-β (Abcam), IL-6 (Abcam), TNF-α (Abcam), Sema3E (Abcam), and β-actin (Santa Cruz Biotechnology). After washing, membranes were incubated with horseradish peroxidase-conjugated (HRP) anti-mouse or HRP anti-goat or -rabbit secondary antibodies (Millipore). Membranes were imaged with LAS-3,000 imager. Protein band intensities were assessed using densitometry plugins in ImageJ and expressed as a ratio between corresponding protein and β-actin.

### Choroidal Sprouting Assay

Choroidal explants were prepared from adult C57BL6/J mice as previously described (Shao et al., 2013). Briefly, eyes were rapidly enucleated and dissected to remove the cornea, lens and retina. The choroid/RPE complex was cut into approximately 1 mm × 1 mm sections and embedded in growth-factor reduced Matrigel in 24-well plates. The choroidal explants are cultured in EBM-2 medium (Lonza, Cat. CC-3156) supplemented with endothelium growth medium (EGM) kit (Lonza, Cat. CC-4147) at 37°C in 5% CO_2_ for 3 days. On day 4, the media was changed to vehicle or recombinant mouse IL-17A (200 ng/ml) in absence or presence of recombinant mouse Sema3E (5 ng/ml), for another 24 h. Phase-contrast photos of individual explants were taken with ZEISS AxioOberver microscope before and 24-h after treatment. The microvascular sprouting area was measured using Image J software version 1.50i (National Institutes of Health, United States).

### Flow Cytometry and Cell Sorting

Freshly dissected retinas from treated-OIR pups were pooled (2 retinas per treatment per n) and digested in HBSS solution containing 750 U/ml DNase I (Sigma-Aldrich) and 1 mg/ml collagenase D (Roche) for 20 min at 37°C with gentle shaking. Homogenized retinas were then filtered through 70 μm cell strainer and supplemented with fluorescent activated cell sorting (FACS) buffer (PBS with 3% FBS) to quench enzyme activity. Cell suspensions were incubated with LEAF purified anti-mouse CD16/32 (Biolegend) for 10 min at 4°C to block Fc receptors, followed by 30 min incubation at 4°C of the following antibodies: FITC anti-mouse CD11b (eBioscience) and APC anti-mouse F4/80 (BioLegend). Dead cells were excluded using 7-AAD viability staining solution (BioLegend). FACS was performed on BD LSRFortessa X-20 and data was analyzed using FlowJo software. Myeloid cells (CD11b^+^ F4/80^+^) were sorted by BD FACSARIA III.

### Statistical Analysis

Results are presented as mean ± SEM for all studies. One-way or two-way analysis of variance with significance α = 0.05 or higher were used for processing data. Bonferroni *post hoc* analysis was used for calculating significance between groups. Two-tailed student *t*-tests were used to test for significance between two means.

## Results

### MSCs Promote Normalization of Ischemic Retinal Vasculature in a Paracrine Fashion

**FIGURE 1:**
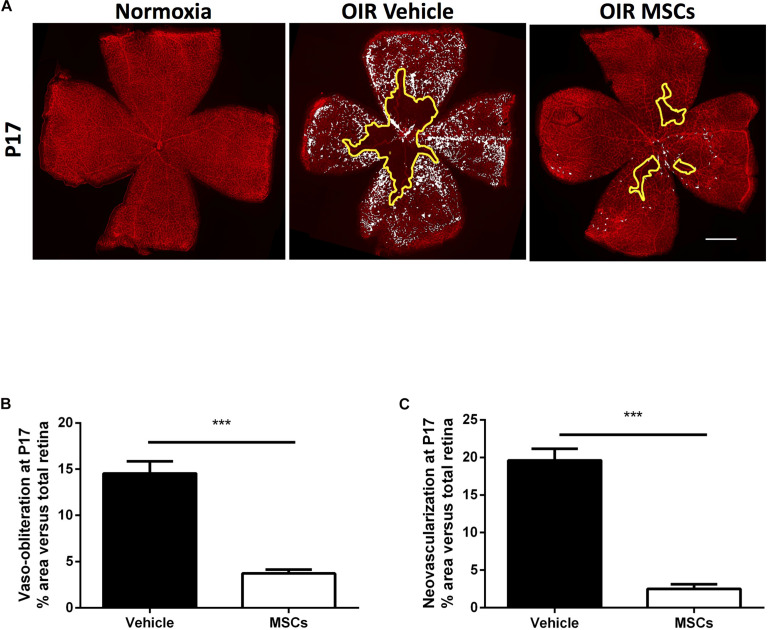
Intravitreal injection of MSCs induced revascularization in OIR retinas. **(A)** Representative photomicrographs from isolectin B4-stained retinal flatmounts from normoxic or OIR mice at P17 intravitreally treated with vehicle or MSCs. Scale bar 500 μm. **(B)** Retinas intravitreally injected with MSCs demonstrate decreased vaso-obliterated (VO) areas (outlined with solid yellow) with respect to OIR vehicle at P17. Quantification of VO areas is shown in graph (***p < 0.001 vs vehicle, values are mean ± SEM, *n* = 6–10 retinas). **(C)** MSCs-treated OIR retinas further show less neovascularization (NV) areas (highlighted in white) compared to vehicle. Quantification of NV areas by *SWIFT_NV* are shown in graphs (***p < 0.001 vs vehicle, values are mean ± SEM, *n* = 6–10 retinas).

**FIGURE 2:**
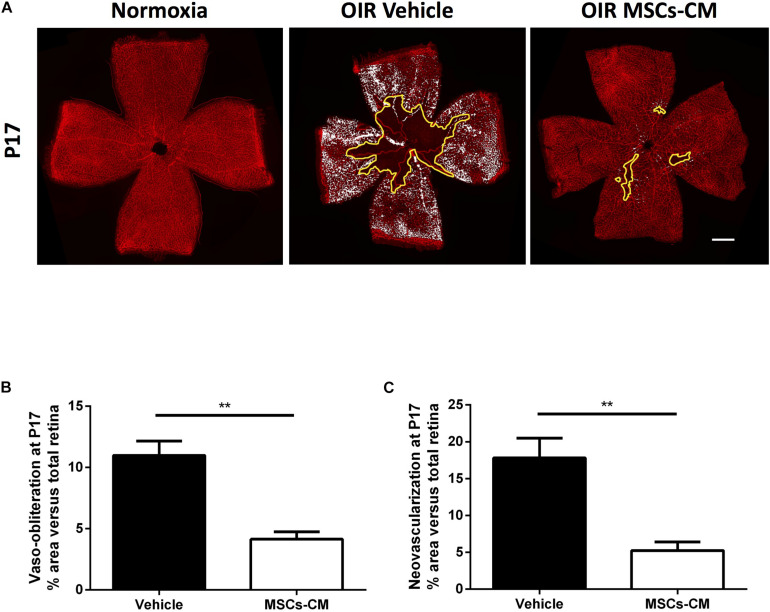
Conditioned media (CM) of hypoxic MSC (MSCs-CM) promoted vascular growth in OIR retinas. **(A)** Representative photomicrographs from isolectin B4-stained retinal flatmounts from normoxic or OIR mice at P17 intravitreally treated with vehicle or MSCs-CM. Scale bar 500 μm. Retinas intravitreally injected with MSCs-CM demonstrate decreased VO **(B)** and NV **(C)** areas highlighted in yellow and white, respectively. Quantification of VO and NV is represented in the graphs (**p < 0.01 vs vehicle, values are mean ± SEM, *n* = 6–10 retinas).

MSCs were intravitreally injected in OIR animals at the beginning of NV phase on postnatal day 12 (P12). At P17, retinas were isolated to evaluate the degree of vaso-obliteration (VO) and consequential pathological NV (Figures 1A–C). Intravitreally administered MSCs [50,000 cells; similar regenerative efficacy to higher cell concentrations (Supplementary Figure 2A)] significantly reduced the extent of VO, thus enhancing revascularization, and consequently diminished pathological NV, compared to naïve media-treated retinas (Figures 1A–C). Although MSCs were effective, occasionally some cells remained clustered at the injection site, while conditioned medium derived from the same number of MSCs (MSCs-CM) reproduced effects of MSCs on retinal angiogenesis and no drawbacks were detected (Figures 2A–C).

### MSCs-Secretome Modulates Retinal Expression of Factors That Affect Retinal Vascular Architectural Network in OIR

**FIGURE 3:**
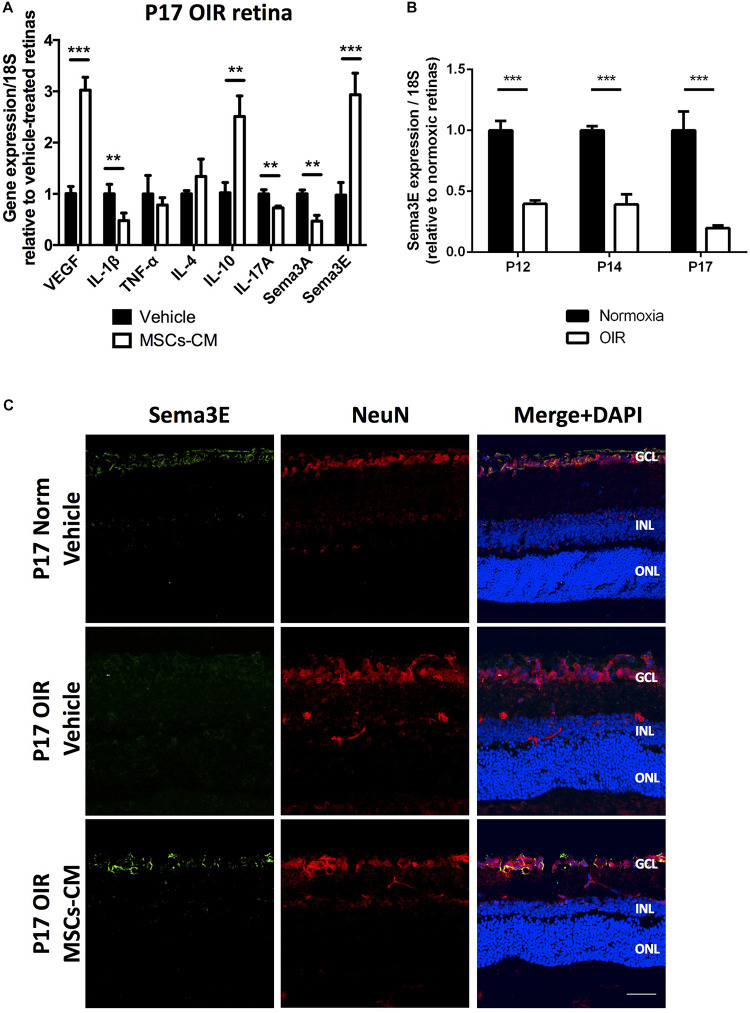
MSCs-CM regulate gene expression and restored Sema3E levels in OIR retinas. **(A)** Real-time quantitative PCR (qPCR) analysis of whole retinas from P17 OIR mice treated with MSCs-CM show, in comparison with vehicle-treated retinas, regulated mRNA levels of growth factor (VEGF), inflammatory cytokines (IL-1β, TNF-α, IL-4, IL-10, and IL-17A), and guidance cues (Sema3A and Sema3E), (**p < 0.01, ***p < 0.001 vs vehicle, values are mean ± SEM, *n* = 4–5, pool of 2 retinas per n). **(B)** Real-time quantitative PCR (qPCR) analysis of whole retinas from mice pups exposed to normoxia or OIR. Sema3E levels consistently decreased following exposure to 75% O_2_ in P12, P14 and P17 pups (*** = *p* < 0.001 vs normoxia, values are mean ± SEM, *n* = 4–5, pool of 2 retinas per n). **(C)** Representative images showing immunohistochemical analysis of P17 mice exposed to OIR (middle panel and vehicle) displayed absence of Sema3E (green) levels in NeuN-expressing retinal ganglion cells (red) in comparison to normoxic retina (top panel, normoxia). Treatment of OIR retinas with MSCs-CM restored neuronal Sema3E levels (bottom panel). Nuclei were counterstained with DAPI (blue). GCL, ganglion cell layer; INL, inner nuclear layer; ONL, outer nuclear layer. Scale bar 100 μm.

A gene expression array of growth (angiogenic), inflammatory and guidance/apoptotic factors (Figure 3A) performed at P17 after OIR revealed in retinas of MSCs-CM-treated animals (at P12) a marked increase in VEGF, a decrease in pro-inflammatory cytokines IL-1β and IL-17A, as well as, an increase in anti-inflammatory IL-10 in comparison to vehicle-treated. These MSCs-CM-induced changes in OIR were associated with a considerable suppression of Sema3A and a notable (three-fold) augmentation of Sema3E which is nearly undetected in untreated OIR retinas at P17 (Figure 3B), consistent with previous reports (Fukushima et al., 2011). The MSCs-CM-induced increase in Sema3E in OIR at P17 was confirmed by RT-PCR (Supplementary Figure 2B), as well as by immunohistochemistry where, as expected (Fukushima et al., 2011; Sharma et al., 2014; Sun et al., 2017), Sema3E was detected in the ganglion cell layer (Figure 3C).

**FIGURE 4:**
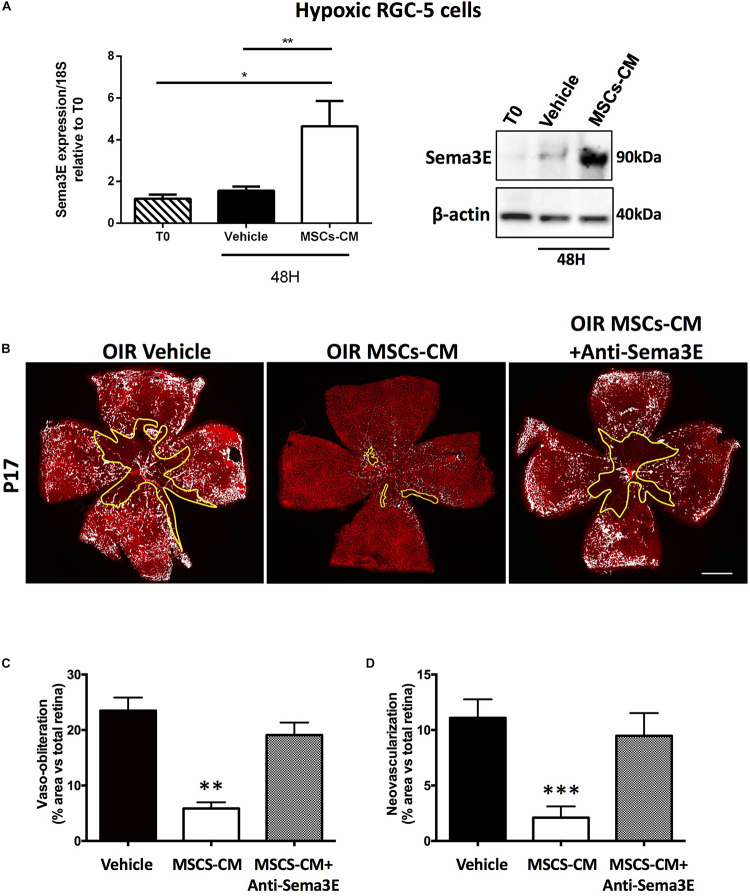
Sema3E is promoted by MSCs-CM in retinal ganglion cells. **(A)** Sema3E levels were evaluated in lysates from RGC-5 cells subjected to MSCs-CM or vehicle for 48 h under hypoxic conditions. No changes were observed on RGC-5 cells treated with vehicle, whereas MSCs-CM treatment upregulated mRNA levels evaluated by qPCR (** = *p* < 0.01, * = *p* < 0.05 vs Vehicle, values are mean ± SEM, *n* = 4–5, pool of 2 retinas per *n*) and protein levels of Sema3E evaluated by Western blot (*n* = 4, pool of 2 retinas per n). **(B)** Representative photomicrographs from isolectin B4-stained retinal flatmounts from P17 OIR mice treated intravitreally with vehicle (PBS) or MSCs-CM in absence or presence of an anti-Sema3E antibody. Scale bar 500 μm. Histograms representing VO **(C)** and NV areas **(D)** that were significantly reduced in MSCs-CM respect to vehicle-treated, but prevented in the presence of the anti-Sema3E (**p < 0.01, ***p < 0.001 vs vehicle, values are mean ± SEM, *n* = 3–4 retinas).

**FIGURE 5:**
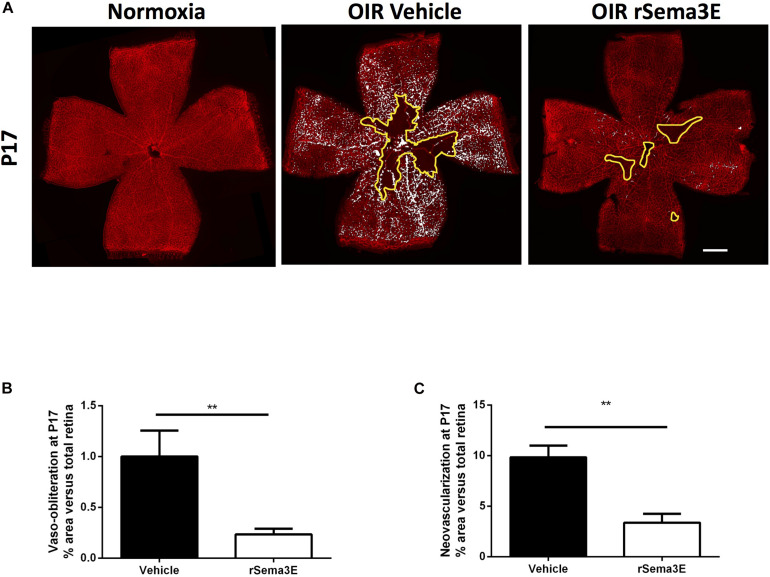
Sema3E promoted vascular regeneration. **(A)** Representative photomicrographs from isolectin B4-stained retinal flatmounts from normoxic or P17 OIR mice treated intravitreally with vehicle (PBS) or recombinant Sema3E (rSema3E). Scale bar 500 μm. Histograms representing VO **(B)** and NV areas **(C)** that were significantly reduced in rSema3E-treated retinas in contrast to vehicle-treated (**p < 0.01 vs vehicle, values are mean ± SEM, *n* = 4–5 retinas).

In an attempt to mimic *in vitro* the *in vivo* conditions related to OIR, we confirmed that MSCs-CM triggered induction of Sema3E gene expression and protein in hypoxic (5% O_2_) RGC (Figure 4A), but not in normoxic (21% O_2_) RGCs (Supplementary Figure 2C). Concordantly, anti-Sema3E prevented the improved retinal vascularization in MSCs-CM-treated mice subjected to OIR (Figures 4B–C). In addition, treatment of mice subjected to OIR with recombinant Sema3E (rSema3E, at P12) markedly curtailed aberrant NV (at P17), in line with a gradual OIR-associated increase in the expression of the Sema3E receptor PlexinD1 (Supplementary Figure 3A); yet surprisingly anti-angiogenic Sema3E also diminished the degree of retinal vasoobliteration (Figures 5A–C).

### Effects of MSCs-CM and Sema3E in OIR Are Associated With Downregulation of Angio-Active IL-17A and Other Proinflammatory Cytokines in Myeloid Cells

**FIGURE 6:**
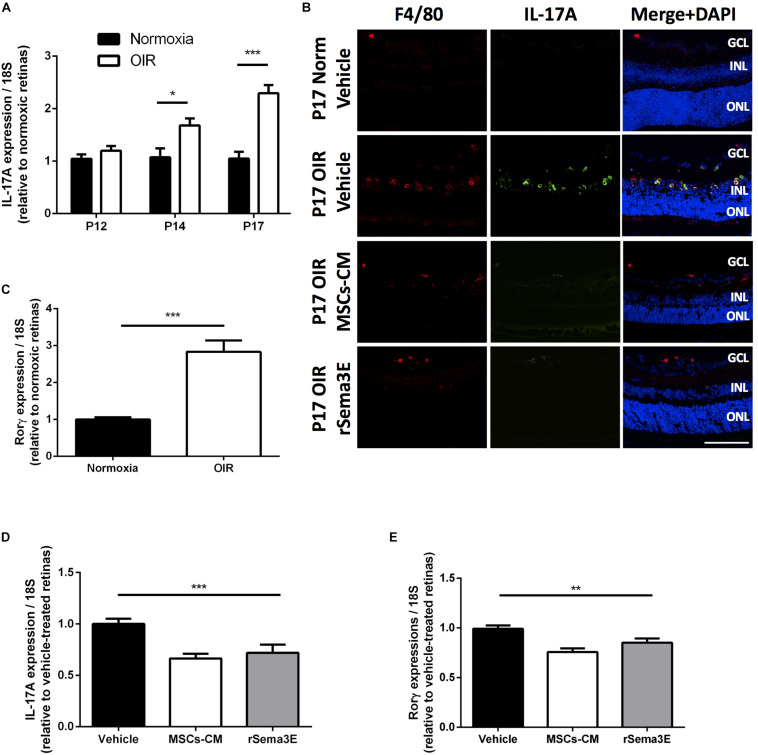
MSCs-CM downregulated IL-17A levels in retinal myeloid cells. **(A)** qPCR analysis of IL-17A in whole retina from mice pups at different time points of OIR demonstrating increased levels of IL-17A mRNA by P14 and P17 (*p < 0.05, ***p < 0.001 vs normoxia, values are mean ± SEM, *n* = 4–5, pool of 2 retinas per n). **(B)** Representative cryosections of normoxic and OIR retinas treated with Vehicle, MSCs-CM or rSema3E demonstrating co-localization of IL-17A with myeloid cell F4/80 marker. Nuclei were counterstained with DAPI (blue). NFL, nerve fiber layer; GCL, ganglion cell layer; INL, inner nuclear layer; ONL, outer nuclear layer. Scale bar 100 μm. **(C)** Real-time quantification (qPCR analysis) of P17 OIR retinas demonstrated increased expression of the nuclear receptor RORγ which regulates IL-17A transcription (***p < 0.001, values are mean ± SEM, *n* = 4–5, pool of 2 retinas per n). **(D)** Intravitreal injection of OIR retinas with MSCs-CM and rSema3E demonstrated lower levels of IL-17A at P17 versus vehicle-injected OIR retinas (***p < 0.001, *n* = 3–4, pool of 2 retinas per n). **(E)** Intravitreal injection of MSCs-CM and rSema3E exhibited significant decreased RORγ expression in OIR retinas via qPCR analysis at P17 in comparison to vehicle-treated counterpart (**p < 0.01, values are mean ± SEM, *n* = 4–5, pool of 2 retinas per n).

As is the case for MSCs (Qian et al., 2020), Sema3E does not only act on endothelial cells (Gu et al., 2005), by inhibiting directly proangiogenic effects of IL-17A (Supplementary Figure 3B), but also exerts effects on myeloid cells (Mohammed et al., 2020). In an attempt to explain opposing actions of Sema3E on vasculature we explored if Sema3E can regulate inflammatory mediators (myeloid cell-derived) which exhibit distinct functions. A lack of Sema3E has been shown to aggravate inflammation (Movassagh et al., 2017a,b) and exacerbate neovascular retinopathy by increasing the release of pro-inflammatory cytokines, particularly the pro-angiogenic IL-17A (Talia et al., 2016). As expected, IL-17A mRNA and protein (immunoreactivity) increased during the neovascular phase starting at P14 (p < 0.05) and furthermore by P17 (*P* < 0.001) (Figures 6A,B); likewise, mRNA expression of the retinoic acid receptor-related orphan nuclear receptor γ (RORγ), a key regulator in the production of IL-17A (Talia et al., 2016), also increased at P17 in OIR (Figure 6C). Intravitreal injection of MSCs-CM or rSema3E reduced significantly mRNA and protein levels of IL-17A, and mRNA expression of RORγ (Figures 6D,E) as well as that of other downstream pro-inflammatory cytokines IL-1β, IL-6, and TNF-α (Supplementary Figure 4A), in OIR retinas evaluated at P17; importantly, contrary to IL-17A, IL-1β elicits retinal vascular degeneration by upregulating Sema3A (Rivera et al., 2013).

**FIGURE 7:**
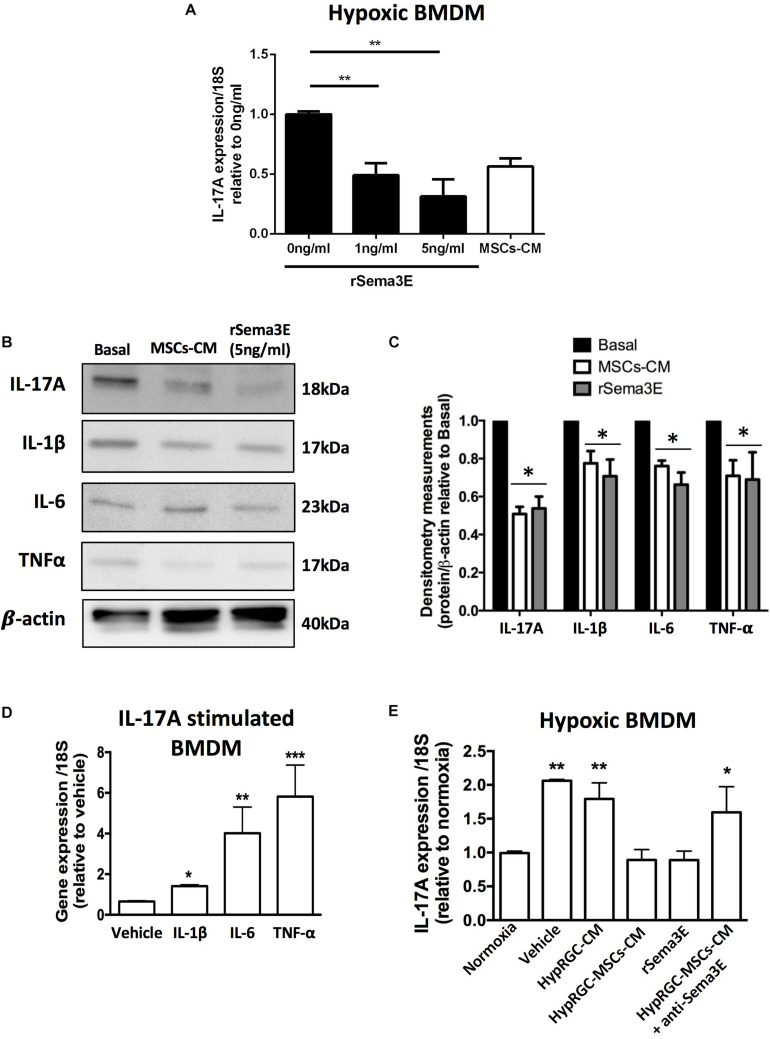
rSema3E regulated myeloid cells-derived IL-17A in a dose-dependent manner. **(A)** Stimulation of hypoxic bone marrow-derived macrophages (BMDM) with rSema3E diminished IL-17A mRNA expression in a dose-dependent manner, while MSCs-CM have a partial effect (**p < 0.01 vs 0 ng/Sema3E, values are mean ± SEM, *n* = 6). **(B)** Representative images from Western blot analysis of pro-inflammatory cytokines (IL-17A, IL-1β, IL-6, and TNF-α) in hypoxic BMDM treated with MSCs-CM and rSema3E (5 ng/ml) showing reduced protein levels of the cytokines with rSema3E and MSCs-CM treatments in comparison to vehicle. β-actin was used as internal control. **(C)** Densitometry quantification at right (* < 0.05 vs Basal, values are mean ± SEM *n* = 3 independent experiments, pool of 2 wells per group). **(D)** Stimulation of hypoxic BMDM with rIL-17A (100 ng/ml) increases IL-1β, IL-6, and TNF-α mRNA expression (*p < 0.05, **p < 0.01, ***p < 0.001 vs vehicle, values are mean ± SEM, *n* = 3). **(E)** Real-time quantitative PCR (qPCR) analysis of bone marrow-derived macrophages (BMDM) pre-exposed to hypoxia (5% O_2_) showing the augmented BMDMs-derived IL-17A expression in vehicle (DMEM alone) and hypoxic RGC conditioned media (HypRGC-CM) treatments after 24 h of incubation. BMDMs treated with the conditioned medium derived from RGCs previously stimulated with MSCs-CM (HypRGC-MSCs-CM) strongly supressed (*p* < 0.01) the mRNA levels of IL-17A in a similar way to the supplementation with the rSema3E (5 ng/ml). The anti-inflammatory effect of HypRGC-MSCs-CM was abrogated in the presence of a neutralizing antibody against Sema3E (*p < 0.05, **p < 0.01, vs normoxia, values are mean ± SEM, *n* = 3).

IL-17A co-localized with F4/80 myeloid cells in the retina, as these are main producers of IL-17A (Talia et al., 2016). Attenuation of the pro-inflammatory cytokine profile of myeloid cells by MSCs-CM and rSema3E, was not related to their density (flow cytometry analysis) (Supplementary Figure 4B), suggesting that MSCs-CM and rSema3E modulate activation state of immune cells rather than infiltration rate of these cells. Correspondingly, we found in PlexinD1-expressing BMDM (Wanschel et al., 2013) that hypoxia-driven IL-17A mRNA expression (Supplementary Figure 4C) was dose-dependently reduced by rSema3E (Figure 7A). Concordant protein expression of IL-17A and of IL-1β, IL-6, and TNF-α was also decreased *in vitro* by rSema3E as well as MSCs-CM (Figures 7B,C), as observed *in vivo* (Supplementary Figure 4A); relevantly, IL-17A induced expression of IL-1β, IL-6, and TNF-α in BMDM (Figure 7D). Correspondingly in retinas of mice subjected to OIR, expression of IL-1β and IL-1β-dependent Sema3A (Joyal et al., 2011; Rivera et al., 2013) were attenuated by anti-IL-17A antibody (Supplementary Figure 5A), consistent with induction of IL-1β by IL-17A (Figure 7D).

Finally, we ascertained a prominent role for MSCs-CM-triggered Sema3E derived from RGC, in suppressing IL-17A mRNA expression in myeloid cells; this effect was abrogated by anti-Sema3E (Figure 7E). Vice versa, the expression of the Sema3E and its receptor was unaffected by IL-17A or anti-IL-17A (Supplementary Figures 5A,B).

## Discussion

Progression of IRs is driven by a complex interplay of factors that activate various signaling pathways involved in dysregulation of vascular, inflammatory, neuronal and metabolic processes (Capitão and Soares, 2016; Rivera et al., 2017a). The ability to target critical factors involved in initial vascular damage and subsequent disoriented vessel formation remains a major challenge for preventing such oculo-vascular diseases. Emerging therapies against IRs often focus on targeting VEGF to ameliorate pathological NV and prevent vision loss; however, anti-VEGF therapy has limited benefits and can exert potential adverse outcomes (Ghasemi Falavarjani and Nguyen, 2013; Ferrara and Adamis, 2016). Alternatively, stem-cell based therapies offer a multifaceted therapeutic approach by targeting multiple underlying pathologic pathways and providing an environment favorable for vascular regeneration. In this study, we harnessed the reparative, regenerative and immunomodulatory potential of MSCs-secretome to promote proper vascular growth in ischemic retinas and prevent pathological neovascular formation. Several studies have shown that the therapeutic effects of MSCs can be associated with soluble paracrine factors and show even better tissue repair than the cells themselves (Uemura et al., 2006; Timmers et al., 2011; Liang et al., 2014; Pankajakshan and Agrawal, 2014; Pokrovskaya et al., 2020). MSCs-secretome has shown a low risk of toxicity, and immune rejection (Baek et al., 2019) making it a promising therapeutic alternative for the treatment of IRs. We investigated the modulatory response of the MSCs-secretome on the expression of key retinal factors involved in vascular repair, notably in enhancing revascularization and thus reducing vasoobliteration. We found that MSCs-CM preventing the development of pathological NV and induced a healthy revascularization in OIR retinas. This beneficial effect of MSCs-CM was associated with a modulation of gene expression of angiogenic, guidance cue and inflammatory factors involved in the progression of retinopathy. Such restoration of growth as well as pro/anti-inflammatory factors, such as VEGF, IL-1, and IL-10, is reported to decrease the occurrence of retinopathy (Hellstrom et al., 2003; Higgins et al., 2003). Moreover, MSCs-CM controlled in opposite directions the expression of two neuron projection repulsive signals, Sema3A and Sema3E, known to exert anti-angiogenic properties, such that MSCs-CM induces Sema3E which in turn suppresses pro- and anti-angiogenic inflammatory factors, which, respectively, curtail pre-retinal NV and facilitate revascularization.

A variety of factors affected by the inherent environment have been identified to partake in reparative and immuno-modulatory properties of MSCs. These include peptide growth factors, lipid mediators, nucleic acids as well as organelles and exosomes (Spees et al., 2016). A key factor presented in this work that partakes in benefits incurred by MSCs applies to Sema3E. Neuronal-derived Sema3E is markedly upregulated by MSCs-CM, and in turn exerts potent anti-inflammatory effects on myeloid cells which are the main producers of potentially detrimental cytokines (Demircan et al., 2006; Mocan et al., 2006; Rivera et al., 2013; Talia et al., 2016) that contribute to vascular injury and pathological NV (Karlstetter et al., 2015). The effects of Sema3E on macrophages are mostly controversial (Shimizu et al., 2013; Wanschel et al., 2013). In this paper MSCs-CM restores Sema3E, which tends to normalize IL-17A concentrations and in turn those of other proinflammatory cytokines, some of which upregulate expression of other Semaphorins, notably Sema3A (Joyal et al., 2011; Rivera et al., 2013). The mechanism by which Sema3E down-regulates IL-17A is possibly through suppression of RORγ, an important regulator of IL-17A (Talia et al., 2016). IL-17A is known to play an important role in aggravating and sustaining local tissue inflammation by enhancing the inflammatory signaling pathways of pro-inflammatory cytokines that impede normal revascularization and enhance pathological NV (Xu and Cao, 2010; McGeachy et al., 2019). In contrast, IL-1β causes retinal vascular degeneration by inducing Sema3A expression (Rivera et al., 2013). The shift in the inflammatory profile of OIR retinas treated with MSCs-CM or rSema3E resulted in a beneficial advantage by favoring an environment suitable for vascular regeneration and dampening preretinal NV in the ischemic tissue.

Although we attribute a significant role for inflammatory mediators IL-17A, IL-1β and possibly IL-6 and TNF-α in the actions of MSCs-CM and Sema3E we cannot rule out the possible contribution of anti-inflammatory mediators, such as angiopoietin-like 4 (ANGPTL4) or IL-10 highly secreted by MSCs (Wang et al., 2018; Cho et al., 2019). Such factors could indirectly modulate Sema3E production through suppressing the secretion of proinflammatory cytokines (Fiorentino et al., 1991; Cho et al., 2019; Sun et al., 2019) which induce ROR-α upregulation and subsequently inhibit Sema3E expression in the retina during pathological conditions. ROR-α is up-regulated by pro-inflammatory cytokines such as IL-1β and TNF-α (Journiac et al., 2009; Cho et al., 2019) and suppresses Sema3E expression in pathological retinal NV (Fukushima et al., 2011; Kim et al., 2011). We can also not exclude an effect of this Semaphorin directly on its receptor PlexinD1 on endothelium (Fukushima et al., 2011; Kim et al., 2011; Suda et al., 2014). PlexinD1 activation promotes filopodial retraction in endothelial tip cells by disrupting integrin-mediated adhesive structures resulting in inhibition of angiogenesis (Sakurai et al., 2010). Its expression is particularly augmented in pathological endothelium as found herein. Altogether, these claims favor PlexinD1 as a viable target in abnormal conditions, relative to VEGF essential in vascular proliferation.

**FIGURE 8:**
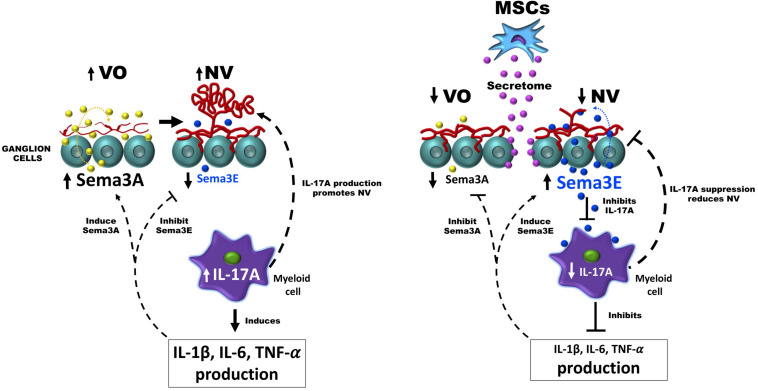
Diagram showing the interplay between neurons and immune cells to facilitate healthy revascularization and diminish abnormal NV in the ischemic retina. During oxygen-induced retinopathy (OIR), the increased production of IL-17A in myeloid cells allows the development of pathological neovascularization (NV), and triggers an increase in the levels of pro-inflammatory cytokines (IL-1β, IL-6, and TNF-α) that stimulate the expression of the anti-angiogenic factor Sema3A and conversely suppress the production of the neuronal factor Sema3E. In the presence of the MSCs secretome, RGCs restore their Sema3E levels that directly inhibit pathological neovascularization through their PlexinD1 receptor located in pathological vessels, or indirectly through supressing the production of angiogenic IL-17A on myeloid cells, which in turn results in a down-regulation of pro-inflammatory cytokines (IL-1β, IL-6, and TNF-α), and of anti-angiogenic factor Sema3A.

In this study, we unveiled an unprecedented interplay between MSCs, neurons and myeloid cells through which MSCs inhibit aberrant NV and promote revascularization in retinopathy, at least in part, by restoring neuronal Sema3E levels and reducing pathological levels of IL-17A in myeloid cells. A limitation in this study is uncovering the identity of MSCs-derived factors that trigger Sema3E production in RGCs. In this regard, MSCs-derived exosomes and microvesicles (Bruno et al., 2015; Rani et al., 2015) that contain a complex set of multiple soluble biologically active molecules could be ideal candidates to regulate Semaphorins in RGCs. The therapeutic efficacy of extracellular vesicles from MSCs has been widely demonstrated in many retinal disease models (Yu et al., 2016; Mead and Tomarev, 2017; Moisseiev et al., 2017; He et al., 2018; Mead et al., 2018; Pan et al., 2019). Due to their nano dimension, extracellular vesicles may rapidly reach and exert their therapeutic effects on RGCs (Harrell et al., 2018) inducing axonal regeneration and improving survival and the maintenance of these cells (Seyedrazizadeh et al., 2020; Usategui-Martin et al., 2020). Notably, microRNAs (miRNAs) as a component of MSCs-derived extracellular microvesicles (Park et al., 2018; Asgarpour et al., 2020) seem to be attractive candidates for semaphorins regulation on RGCs. miRNAs have been shown to modulate semaphorin expression, for instance, miR-497-5p is a critical target of Sema3A (Shapoori et al., 2019) while miRNA-4282 has been shown to be a key regulator of Sema3E expression (Kang et al., 2016). Determining the presence of these miRNAs as part of MSCs-secretome could be of great relevance for evaluating its action on RGCs. Exosome-derived lipids could be other candidates for regulating semaphorins in RGCs. Exosomes contain large amounts of cholesterols (de Gassart et al., 2003; Deng et al., 2018). Cholesterol derivatives such as 7α-hydroxycholesterol, 7β-hydroxy-cholesterol and 7-keto-cholesterol are natural ligands and negative regulators of the nuclear receptor ROR-alpha (Solt and Burris, 2012), responsible for inhibiting the expression of Sema3E in RGCs during OIR (Sun et al., 2017). Although we do not rule out a possible action of MSCs-derived cytokines/growth factors on RGCs, future work will certainly be aimed at evaluating the MSCs-CM-derived extracellular vesicle elements involved in the regulation of semaphorins in RGCs. So far, our study shows the ability of MSCs-CM to promote the production of neuronal factors involved in immunomodulatory activities which would generate a microenvironment permissive for vascular regeneration, and thus offer a promising opportunity for the treatment of IRs using stem cell therapy. Collectively, we show evidence by which MSC secretome regulates the interplay between neurons and immune cells establishing a healthy environment permissive for vascular regeneration in ischemic retina; a schematic diagram depicting the described mode of action of MSCs secretome in ischemic retinopathy is presented in Figure 8.

## Data Availability Statement

The original contributions presented in the study are included in the article/Supplementary Material, further inquiries can be directed to the corresponding author/s.

## Ethics Statement

The animal study was reviewed and approved by the Animal Care Committee of Maisonneuve-Rosemont Hospital in accordance with guidelines established by the Canadian Council on Animal Care and the Association for Research in Vision and Ophthalmology (ARVO) statement for the use of animals in ophthalmic and vision research.

## Author Contributions

BN, SC, and JCR conceived and designed the study and wrote the manuscript. BN, JCR, RD, PA, SO, and IL performed the experiments. BN and JCR prepared the figures. JCR designed the scheme. All authors contributed to the article and approved the submitted version.

## Conflict of Interest

The authors declare that the research was conducted in the absence of any commercial or financial relationships that could be construed as a potential conflict of interest.
