# Supplementary material for: Mesenchymal Stromal Cells Promote Retinal Vascular Repair by Modulating Sema3E and IL-17A in a Model of Ischemic Retinopathy
Source: Front Cell Dev Biol. 2021 Jan 21;9:630645. doi: 10.3389/fcell.2021.630645 (PMC7859341; doi:10.3389/fcell.2021.630645)
Supplement: Supplementary file 1 [file Data_Sheet_1.docx]

Supplementary Material

## Supplementary Figure 1

**Supplementary Figure 1. Characterization of MSC from mouse compact bone. (A)** Representative images from flow cytometry quantification of MSCs demonstrated abundant expression of the positive markers CD90, CD105 and CD73. **(B)** Representative images showing flow cytometry quantification of MSCs exhibited negligible expression of the myeloid marker CD11b and hematopoietic stem cell marker CD34. **(C)** Representative microscopy images of differentiated MSCs into adipocytes by FABP4 staining (red, left panel) and osteocytes by oseteopontin staining (red, right panel). FABP4=Fatty Acid-Binding protein 4.

## Supplementary Figure 2

**Supplementary Figure 2. Intravitreal administration of hypoxic MSCs cells reduces vasoobliteration, while MSCs-CM increases production of Sema3E in OIR retinas at P17. (A).** Intravitreal administration of 50, 000 MSCs significantly reduced VO areas in OIR at P17 (p<0.001 vs Vehicle, values are mean ± SEM, n=4-5) to similar extent as higher cell doses (100, 000 and 200,00). **(B)** MSCs-CM-treated OIR retinas show increased Sema3E expression at P17. (***p<0.001 vs vehicle, values are mean ± SEM, n=4-5, pool of 2 retinas per n). **(C)** Real-time quantitative PCR (qPCR) analysis on Sema3E levels evaluated in RGC-5 cells subjected to vehicle or MSCs-CM for 48 hours under normoxic conditions. No changes on Sema3E mRNA levels were observed on RGC-5 cells treated with vehicle, or MSCs-CM (Values are mean ± SEM, n=4-5).

## Supplementary Figure 3

**Supplementary Figure 3. PlexinD1 expression levels in the retina during neovascularization phase. (A)** Real-time quantitative PCR (qPCR) analysis of OIR versus normoxic retinas indicated a steady increase of PlexinD1 levels, which is selectively expressed by neovascular tufts. (*p<0.05, **p<0.01, ***p<0.001, values are mean ± SEM, n=4-5, pool of 2 retinas per n). **(B)** Representative images from choroidal explants exposed to basal media or rIL-17A (200 ng/ml) in presence or absence of rSema3E (5ng/ml) for 24 hours. rSema3E significantly suppresses vascular sprouting induced by rIL-17A. Scale bar 200μm. The graphs represent the quantification of sprouting area (**p<0.01 vs Vehicle * p<0.05 vs rIL-17A, values are mean ± SEM, n=6-8).

## Supplementary Figure 4

**Supplementary Figure 4. The production of proinflammatory cytokines rather than that the density of myeloid cell is affected by treatment with MSCs-CM or rSema3E. (A)** Real-time quantification of different pro-inflammatory cytokine (IL-1β, IL-6, TNF-α) from retinal myeloid cells (CD11b+ F4/80+) sorted from vehicle, MSCs-CM, and Sema3E-treated retinas. MSCs-CM and rSema3E-injected retinas significantly downregulated the cytokines’ expression (**p<0.01, ***p<0.001 vs vehicle, values are mean ± SEM, n=3-4). **(B)** Representative images from flow cytometry analysis of CD11b+ F4/80+ myeloid cells from P17 OIR retinas. Intravitreal injection of MSCs-CM or rSema3E did not change the myeloid cell density compared to vehicle treatment. Quantification at bottom. (ns=non-significant, n=6-10). **(A)** Real-time quantitative PCR (qPCR) analysis of BMDM exposed to normoxia (21% O_2_) or hypoxia (5% O_2_). Following 24-hour exposure to hypoxia, BMDM overexpressed the pro-inflammatory cytokine IL-17A. (**p<0.01 vs Normoxia, values are mean ± SEM, n=3).

## Supplementary Figure 5

**Supplementary Figure 5. IL-17A antibody suppresses retinal expression of pro-inflammatory mediators in vivo, while IL-17A does not regulate Sema3E expression in RGC-5 cells. (A)** Real-time (qPCR analysis) of P17 OIR retinas treated with Neutralizing IL-17A antibody showed decreased expression of IL-17A, IL-1β and Sema3A expression without affecting that of Sema3E and its receptor PlexinD1 (*p<0.05 vs IgG, values are mean ± SEM, n=3, pool of 2 retinas per n). **(B)** Exposure of RGC-cells to IL-17A in a dose-dependent manner does not promote Sema3E secretion in normoxic or hypoxic conditions (values are mean ± SEM, n=3-4).
